# Characterization of antibodies against the replication protein (Rep) encoded by bovine meat and milk factors (BMMFs)

**DOI:** 10.1007/s00253-026-13809-x

**Published:** 2026-04-16

**Authors:** Veronika Frehtman, Gunjan Shukla, Michael Gentz, Marcus Müller, Oladimeji Paul Duduyemi, Imke Grewe, Claudia Ernst, Claudia Tessmer, Andrea Didier, Ilse Hofmann, Timo Bund, Barbara Leuchs

**Affiliations:** 1 German Cancer Research Center, Biopharmaceutical Processing and Development Unit, Im Neuenheimer Feld 280, 69120 Heidelberg, Germany; 2 German Cancer Research Center, Episomal-Persistent DNA in Cancer- and Chronic Diseases, Im Neuenheimer Feld 280, 69120 Heidelberg, Germany; 3 German Cancer Research Center, Monoclonal Antibody Unit of the Genomics and Proteomics Core Facility, Im Neuenheimer Feld 280, 69120 Heidelberg, Germany; 4 Faculty of Veterinary Medicine, Ludwig Maximilians University Munich, Schönleutnerstraße 8, 85764 Oberschleißheim, Germany

**Keywords:** Bovine meat and milk factor (BMMF), Antibody characterization, Antibody specificity, ELISA

## Abstract

**Abstract:**

Bovine Meat and Milk Factors (BMMFs) are DNA elements with similarity to bacterial plasmids, are frequently identified in bovine meat and milk and were proposed to contribute to cancer development. All known BMMFs encode a conserved replication protein (Rep), allowing for histologic BMMF detection in clinical specimens based on Rep-directed mouse monoclonal antibodies (mAbs), which, however, have only been partially characterized so far. Here, 20 anti-BMMF Rep antibodies were assessed for biophysical properties, reactivity, specificity and binding sensitivity to five distinct BMMF Reps and other prokaryotic/eukaryotic target antigens using an enzyme-linked immunosorbent assay (ELISA)-based anti-BMMF Rep antibody binding assay. We demonstrated sensitive and specific antibody reaction with their respective Rep targets, according to the antibody immunization. Consensus antibodies raised against defined peptides of conserved Rep amino acid stretches interacted with most of the Rep antigens. Antibodies produced based on immunization with the Rep encoded on the BMMF isolate H1MSB.1, including rabbit and human chimeric variants, reacted only with the cognate H1MSB.1 Rep, with only two outliers targeting additional Reps. Completely new antibodies raised against the Rep of another isolate (C1HB.4) specifically detected the cognate C1HB.4 Rep antigen – not interacting with other Reps. New antibodies generated by triple Rep immunization (H1MSB.2/C1MI.3M.1/C1MI.9M.1 Rep) reacted to either all three or two immunization antigens without interacting with any other Reps. None of the antibodies cross-reacted against Reps of bacteria occurring during milk production or lysates of mammalian hosts. Competitive inhibition confirmed antigen-specificity across the antibody panel, which additionally did not show aberrancies concerning purity or antibody size for the majority of the tested Abs. These findings authenticate a highly specific panel of anti-BMMF Rep antibodies, which can serve as tools for BMMF detection in cancer and chronic diseases.

**Key Points:**

*• Anti-BMMF Rep antibodies are important to judge BMMFs' role as cancer risk factors.*

*• Selective binding of anti-BMMF Rep antibodies to BMMF Rep antigens.*

*• No cross-reactivity of anti-BMMF Rep antibodies with bacterial and mammalian outgroup specimens.*

**Supplementary Information:**

The online version contains supplementary material available at 10.1007/s00253-026-13809-x.

## Introduction

According to epidemiological studies, around 15–20% of cancers are caused by infectious agents (de Martel and Franceschi 2009; de Martel et al. 2020; zur Hausen 2009). Among the more recently described cancer risk factors are Bovine Meat and Milk Factors (BMMFs), which are bacterial plasmid-like DNA sequences frequently isolated from dairy and meat products (de Villiers et al. 2019). Current evidence suggests that BMMFs may function as biomarkers for cancer and potentially act as dietary cancer risk factors. Several immunohistochemical (IHC) studies using monoclonal antibodies directed against BMMF replication (Rep) proteins reported elevated detection levels of BMMF Rep proteins in tissues of cancer patients, in particular suffering from colorectal cancer (CRC), when compared to tissues of healthy non-cancer individuals and therefore suggest a role of BMMFs as biomarkers and application of the antibodies in diagnostic approaches. BMMFs have been supposed to be implicated in the indirect carcinogenesis of CRC upon observation of chronic inflammation of BMMF-affected mucosal tissue, radical formation, and DNA damage in proliferating cells, which can fuel progression to adenoma and carcinoma (Bund et al. 2021; zur Hausen et al. 2017, 2019). Subsequent studies have also reported BMMF Rep expression in hepatocellular carcinoma and colorectal liver metastases, suggesting a broader potential involvement of BMMFs in the context of inflammation-associated carcinogenesis (Nikitina et al. 2024; Siqin et al. 2025).

Currently, more than 200 BMMFs have been described and classified into four distinct BMMF groups (BMMF1-4) based on their sequence homology (de Villiers et al. 2019; Habermann et al. 2023; Koenig et al. 2021; Szigeti-Buck and Manuelidis 2019). Several BMMF1 representatives, including H1MSB.1, which was originally isolated from multiple sclerosis (MS) brain tissue but was frequently isolated from IHC-positive CRC tissue regions, in addition, demonstrated in vitro replication, transcription, and expression in human cells (Eilebrecht et al. 2018). BMMF1 isolates encode a conserved replication initiator protein (Rep), (de Villiers et al. 2019). Monoclonal antibodies (mAbs) generated against either full-length Rep proteins or consensus peptides covering conserved motifs across several Reps of BMMF1 isolates have enabled histological, tissue-based characterization of Rep expression, which was increased in cancer tissues. This suggested a role of BMMFs as potential dietary biomarkers that might contribute to cancer formation (Bund et al. 2021; Nikitina et al. 2024; Siqin et al. 2025).

Detection of BMMFs in clinical samples has relied predominantly on immunohistochemical, antibody-based methods for BMMF protein detection. However, these readouts depend heavily on antibody specificity, especially of the most frequently applied antibody mAb3. Previous studies characterized a set of 14 BMMF1 antibodies (mAb1–11, 13–15) against a limited set of antigens, including mapping of linear epitopes and demonstrating specificity across western blot (WB), ELISA, and immunofluorescence assays (Bund et al. 2021). However, in the meantime, additional potential cancer-related BMMF isolates have been identified in cancer patients based on classical DNA isolation as well as in cancer sequencing data including BMMF1 isolates C1HB.4, H1MSB.2, C1MI.3M.1 and C1MI.9M.1 (Bund et al. 2021; de Villiers et al. 2019; Häfele 2024; Siqin et al. 2025). Besides, questions about the proximity of BMMFs to bacterial plasmids (de Villiers et al. 2019; Kilic et al. 2019), which might potentially represent BMMF origins, prompted us to extend the panel of BMMF antibodies and their potential repertoire of detectable antigens. Therefore, we expanded the set of available BMMF antibodies by four new antibodies raised against the newly identified targets (mAb20 and mAb21 against C1HB.4 Rep, mAb22 and mAb23 against H1MSB.2, C1MI.3M.1 and C1MI.9M.1 Rep) as well as a rabbit and a human chimeric variant of mAb3. A newly established enzyme-linked immunosorbent assay (ELISA)-based anti-BMMF Rep antibody binding platform facilitated a side-by-side comparison with previous antibodies, revealing a sensitive and specific reaction of all 20 tested antibodies to five different antigens. Antibody characterization confirmed the expected single target-specific, group-based or consensus-like reactivity to five distinct BMMF Reps, including authentication of specificity and binding sensitivity without cross-reactivity with bacterial Rep or other outgroups.

## Materials and methods

### Antibody generation and purification

Mice (BALB/c or C57BL/6N strain, Charles River Laboratories, Freiburg, Germany) were immunized with either denatured, affinity-purified full-length C1HB.4 Rep or a mixture of full-length H1MSB.2, C1MI.3M.1, C1MI.9M.1 Rep protein (20 µg per immunization). Immunization was administered via subcutaneous injection over 14 days with four injections for the full-length C1HB.4 Rep or over 75 days with repeated immunizations of the Rep mixture. Seropositivity of peripheral blood samples of the different mice was verified by WB on purified Rep protein as antigen. For each immunization setup, the animals showing the highest band intensities in WB were selected for hybridoma fusion according to the principles of Koehler and Milstein's hybridoma technology (Koehler and Milstein 1975). The supernatants were screened based on detection of HEK293TT-expressed Rep by immunofluorescence (IF) and WB (with purified antigens) as well as IHC with cancer tissues. Mother clones with confirmed reactivity were subcloned by cell spotting with a single cell dispenser (F.SightTM, Cytena, Freiburg, Germany). The immunoglobulin isotype was identified by a sandwich ELISA using a pan mouse antibody for catching (goat anti mouse IgG + IgM (H + L), Dianova/Biozol, Hamburg, Germany) and horseradish peroxidase coupled, subclass specific antibodies for detection (goat anti mouse IgG1, Dianova/Biozol, Hamburg, Germany; goat anti mouse IgG2a, Dianova/Biozol, Hamburg, Germany, goat anti mouse IgG2b, Dianova/Biozol, Hamburg, Germany, goat anti mouse IgG2c, Cell Signaling Technology Europe, Leiden, Netherlands, goat anti mouse IgG3, Dianova/Biozol, Hamburg, Germany, goat anti mouse IgM, Dianova/Biozol, Hamburg, Germany).

For generation of antibodies from subcloned hybridomas, supernatants were filtered (0.45 µm, Neolab Migge, Heidelberg, Germany) and the purification of antibodies was carried out using standard affinity chromatography techniques, employing protein A sepharose beads for mouse and rabbit antibodies, and protein G sepharose for human antibodies. Antibodies were eluted with acidic buffer (100 mM glycine, pH 6.0 for mAb20 and mAb21 or 100 mM glycine, 100 mM NaCl, pH 3.2 for mAb22 and mAb23), followed by neutralization with a basic stock solution (1 M Tris–HCl, pH 9.0) and by dialysis overnight against PBS at 4 °C.

For the hybridoma of H1MSB.1-Rep immunized mAb3, RNA was isolated and sequenced to identify the variable heavy (VH) and kappa light (VL) chains using the MiXCR software (Bolotin et al. 2015). The sequences were cloned in frame into two independent human or rabbit backbone vectors, coding for the constant heavy (CH) and kappa light (CL) chains (Wardemann and Busse 2019) respectively. Finally, polyethylenimine (PEI)-mediated transient transfection of FreeStyle-293F cells was applied (PEI branched, ~ 25 kDa, Sigma-Aldrich, Merck, Darmstadt, Germany) with expression plasmids allowing the production of H1MSB.1-ChAb (hAb3 and rAb3), which were composed either of a human or rabbit constant domain and the mouse variable regions of the heavy and light chain. Supernatants were harvested when cell viability dropped below 60% as measured by trypan blue staining (Countess automated Cell counter, Invitrogen, Thermo Fisher Scientific, Munich, Germany). Supernatants were filtered (0.45 µm, Neolab Migge, Heidelberg, Germany) and purified by affinity chromatography with protein A (rAb3, elution in 100 mM glycine, pH 2.7, neutralization with 1 M Tris–HCl, pH 9.0) or protein G (hAb3, elution in 100 mM glycine, pH 2.0, neutralization with 2 M Tris–HCl, pH 8.0) followed by dialysis overnight against PBS at 4 °C.

### Production of BMMF Rep proteins and peptides

*Escherichia coli* SoluBL21 cells (Genlantis/Amsbio, Alkmaar, Netherlands, 50 µl) were chemically transformed with 10 ng of the respective BMMF Rep expression plasmids (Table 1), prior to addition of 400 µl LB medium and incubation for 1 h at 37 °C. 10 µl and 100 µl of the transformation were plated on two separate LB agar plates with 0.1 mg/ml ampicillin (LB-Amp) and incubated overnight at 37 °C. After three rounds of single clone selection, glycerol stocks of transformed bacteria were prepared with an OD_600_ of > 0.6 and < 0.8 and frozen at −80 °C. The BMMF Rep protein was produced by adding 2 µl of bacteria glycerol stock per 1 ml LB-Amp media and incubating the solution at 110 rpm and 37 °C (incubator shaker Innova 4200, New Brunswick, Hamburg, Germany) until an OD_600_ of 0.3–0.4 was reached. After 4 °C overnight storage, the bacteria were incubated until they reached an OD_600_ of 0.6–0.8. The bacteria suspension was induced with 0.66 mM isopropyl β-D-1-thiogalactopyranoside (IPTG, Carl Roth, Karlsruhe, Germany) and further incubated until an OD_600_ of 1.5–1.7 followed by centrifugation (2500 g). The pellet was lysed either in 100 mM sodium carbonate including 163 mM NaCl with 1.5% Triton X-100, pH 9.6 or in PBS including 163 mM NaCl with 1.5% Triton X-100, pH 7.4, clarified with 1.2 µm and 0.1 µm filter (Sartorius, Göttingen, Germany) and purified by a nickel metal affinity chromatography (IMAC, His60 slurry, TaKaRa Bio, San Jose, CA, USA). The final formulations were 100 mM sodium carbonate 300 mM imidazole, pH 9.5 (NaCa) or PBS with 300 mM imidazole, pH 7.2 (PBS). The protein content was measured. Peptide 3 was used to confirm epitope of mAb22 and mAb23. All peptides were produced at Peptide Specialty Laboratories GmbH, Heidelberg, Germany. All proteins and peptides were stored at 4 °C.

**Table 1:** Information on antigens used in the anti-BMMF Rep antibody binding assay, including their origin, sequence information, and characteristics of the expression systems

Antigen	Origin (BMMF isolate, other)	GenBank ID (DNA)	GenBank ID (Rep)	Position of ORF (nt)	Length of antigen (amino acids) (Rep + tag)	Tag for affinity purification	Plasmid used for expression	Promoter type	Codon optimization	Reference/sequence
H1MSB.1 Rep	H1MSB.1	LK931491	CDS63398.1	602–1576	332	KG HHHHHH	pEXP-CT/TOPO	T7	No	10.1128/genomeA.00849–14
H1MSB.1 WH1 Rep				602–1009	168					10.1128/genomeA.00849–14
H1MSB.1 WH1 + WH2 Rep				602–1285	260					10.1128/genomeA.00849–14
H1MSB.1 WH2 + C-term Rep				1010–1576	221					10.1128/genomeA.00849–14
H1MSB.2 Rep	H1MSB.2	LK931492	CDS63399.1	603–1562	327	KG HHHHHH	pEXP-CT/TOPO	T7	No	10.1128/genomeA.00849–14
C1MI.3M.1 Rep	C1MI.3M.1	LR215499	CDS63399.1	292–1260	328	RS HHHHHH	pQE60	T5	Yes	10.1080/22221751.2019.1651620
C1MI.9M.1 Rep	C1MI.9M.1	LR215496	VEV85366.1	297–1262	329					10.1080/22221751.2019.1651620
C1HB.4 Rep	C1HB.4	LK931496	CDS63406.1	480–1406	318					10.1128/genomeA.00846–14
Rep peptide 1	BMMF1			695–748*	18	-	-	-	-	EARETGKGINANDPLTVH
Rep peptide 2	BMMF1			1188–1249*	21	-	-	-	-	KQINEHTDITASYEQHKKGRT
Rep peptide 3	H1MSB.2	LK931492	CDS63399.1	1515–1559	15	-	-	-	-	IFTALKTETDYSKKN
recombinant *E. coli* RepA	Commercial									Cusabio, USA, CSB-PA706568XA01ENL

Codon-optimization of the Rep genes in the expression plasmids was prioritized for human (*Homo sapiens*) and prokaryotic (*E. coli*) codon usage/expression. Rep peptides 1 and 2 correspond to amino acid regions conserved among several Rep proteins of BMMF group 1 (* indicates nucleotide positions in the H1MSB.1 sequence as a reference). Peptides 1 and 2 were also used for individual immunization of mice to generate consensus antibodies (Bund et al. 2021). Rep peptide 3 was synthesized based on amino acids 305–319 of the H1MSB.2 Rep protein (nt 1515–1559 of the Rep open reading frame [ORF])

### Protein and IgG quantification

The protein concentration of antibody or BMMF Rep preparations was analyzed by Bradford assay using bovine serum albumin (BSA) standard (Thermo Scientific, Waltham, MA, USA) in a concentration of 2.5–100 µg/ml. Minimal sample dilution was 1:5 in the respective buffer. For the assay, 50 µl standard or sample were placed in a 96-well plate (Maxisorp, Thermo Scientific, Waltham, MA, USA) and 200 µl 1 × Roti® Nanoquant (Carl Roth, Karlsruhe, Germany) were added, followed by 5 min incubation at room temperature (RT). Evaluation was done by measurement of the absorbance A_450nm_ and A_590nm_ with a plate reader (FLUOstar Omega, BMG Labtech, Ortenberg, Germany) and using the ratio of A_590nm_/A_450nm_, according to the Roti® Nanoquant protocol, for further analyses. The mean concentration was calculated based on triplicates.

The IgG concentration was quantified depending on the IgG subtype with either mouse IgG ELISA Flex kit (Mabtech, Nacka Strand, Sweden) for antibodies 1–11, 13–15 and 20–23 or human IgG high sensitivity ELISA Flex kit (Mabtech, Nacka Strand, Sweden) for hAb3, according to manufacturers’ instructions using kit standards and dilution buffer (PBS with 0.05%Tween and 0.1% BSA) as a background.

### Dynamic light scattering (DLS)

The samples were diluted to similar protein concentration in a linear range of 0.1 to 0.3 mg/ml and loaded into capillaries (NanoTemper Technologies, Munich, Germany), which were placed into a Prometheus Panta device (NanoTemper Technologies, Munich, Germany), and the size distribution (mean of 10 measurements) was assessed with “*Panta control*” software. The size distribution was evaluated by a combination of hydrodynamic radius of the sample and its relative frequency (intensity distribution), where the average scattering intensity is the sum of the detected intensity fluctuations over time and proportional to the size and concentration of particles. To distinguish background noise from a valid measurement, any peak with a relative frequency < 2% is neglected.

### Antibody epitope mapping

Epitope mapping was performed using peptide microarrays containing 15-mer linear peptides (14 amino-acid overlap) spanning either the C1HB.4 Rep sequence (mAb20 and mAb21) or the Reps of H1MSB.2, C1MI.3M.1, and C1MI.9M.1 (mAb22 and mAb23) (PEPperPRINT, Heidelberg, Germany). Each peptide was printed in duplicate peptide spots to allow internal assessment of signal reproducibility. Peptide arrays were equilibrated in staining buffer (PBS, 0.05% Tween-20, 10% blocking reagent) for 15 min at RT prior to exposure to purified monoclonal antibodies (antibody incubation with 1, 10 and 30 µg/ml for C1HB.4 and 1, 10 and 50 µl for the triple-Rep chips) in staining buffer (PBS, 0.05% Tween-20, and 10% blocking reagent; 100 µL total incubation volume per chip, overnight incubation at 4 °C with gentle agitation). Arrays were subsequently washed three times in standard buffer (PBS containing 0.05% Tween-20, pH 7.4) and incubated with anti-mouse IgG secondary antibody (anti-Mouse IgG DyLight680, 0.2 µg/ml, Dianova, Hamburg, Germany) in staining buffer, 45 min, RT. After washing, fluorescence signals were acquired using a LI-COR Odyssey® scanner (LI-COR Biotechnology – GmbH, Bad Homburg, Germany), mapped to the respective Rep sequences and analyzed using the manufacturer’s software (Mapix 9.1.0, Innopsys, Chicago, IL, USA). Putative epitope stretches with consistently high fluorescence detection (at least a factor 1000 over the secondary antibody reference background intensity), over at least 5 consecutive 15-mers and conserved over the three different concentrations for primary antibody incubation were extracted manually and tested by ELISA with synthetic peptides for confirmation of epitopes.

### Anti-BMMF Rep antibody binding assay (ELISA)

200 ng BMMF antigen, 1 µl sera or bacterial lysate was diluted in 100 µl PBS buffer or NaCa buffer (for antigen in NaCa formulation) followed by coating on 96-well Maxisorp plate (Thermo Scientific, Waltham, MA, USA) at 4 °C overnight, blocked in PBS 0.05% Tween-20 (0.05% PBST) with 10% BSA for 1 h at RT (22 ± 1 °C), washed five times with 0.05% PBST before primary antibody addition for 1 h of incubation at RT and another five washing cycles. The primary antibodies 1–11, 13–15 and 20–23 were diluted 1:100 to 1:1000 except for hAb3 and rAb3, which were diluted 1:500.000 and 1:50.000, respectively. After incubation with the detection Ab (rabbit anti-mouse IgG HRP, 1:2500, Rockland, Pottstown, PA, USA, goat anti-human IgG HRP, 1:10,000, or anti-rabbit HRP, 1:2500, both Jackson ImmunoResearch, Cambridge, UK) in 0.05% PBST for 1 h at RT and three times washing, TMB super slow substrate was added (Sigma Aldrich, St. Louis, MO, USA) and colorimetric reaction terminated with 1 M sulfuric acid prior to measurement of the absorbance A_450nm_ (and A_595nm_ as a control for plate imperfections) with a plate reader (FLUOstar Omega, BMG Labtech, Ortenberg, Germany). Antibody binding signal was defined as A_450nm_-A_595nm_.

For the assay development, BMMF Rep antigen coating concentrations ranging from 0.1 to 18 µg/ml were tested for calibration (Supplemental Fig. S1, A). An antigen coating concentration of 2 µg/ml has been determined to be optimal and was thus employed routinely, unless stated otherwise. A range of antibody concentrations was assessed to identify the interval of linear response, yielding antibody binding signals between 0.3 and 2.5 (A_450nm_-A_595nm_) (Supplemental Fig. S1, B). To evaluate the assay performance and its robustness, technical assay controls were included in each assay to ensure its reproducibility and quality. The negative control antibody binding signal was close to or below 0.1 across all experiments and antibodies, demonstrating no interaction with the corresponding matrix (background) (Supplemental Fig. S1, C)._._ The cutoff for discrimination of antibody reactivity was defined as three-fold the background, with a binding signal of ≥ 0.3. Furthermore, the effects of the sample and secondary antibody, alone, were assessed, and sample/matrix spiking with defined amounts of BMMF Rep was performed to rule out assay interference or inhibition. Each assay implemented technical, antibody and negative controls. Samples were analyzed at least in duplicate with representation of the mean value of the binding signal. The anti-penta His antibody (Qiagen, Hilden, Germany) was used for detection of all His-tagged antigens and mouse IgG1 isotype control antibody (Invitrogen, Karlsruhe, Germany) was used as negative control.

### Definition of parameters for characterization of anti-BMMF Rep antibodies

The following terms and parameters have been defined to accurately describe specific properties of BMMF antibodies.

*Purity* of antibodies is defined as the ratio of the IgG concentration of the antibody preparation determined by ELISA to the protein concentration of the same preparation determined by Bradford assay.

$$Purity (\%)= \frac{IgG concentration [\frac{mg}{ml}] \times 100\%}{Protein concentration [\frac{mg}{ml}]}$$

*Reactivity* of an antibody to a specific antigen was defined whenever the antibody binding signal exceeded absorptions of ≥ 0.3 (A_450nm_^−^ A_595nm_) for a given antigen.

*Specificity* demonstrates how effective an antibody detects and distinguishes between antigens that might be structurally or sequentially similar to the tested BMMF Rep protein (EMA 2022). To assess specificity, the unspecific cross-reactivity of all antibodies to bovine and mammalian sera, cells and protein, as well as to the prokaryotic host cell protein lysate is evaluated by anti-BMMF Rep antibody binding assay (see “Cross-reactivity of antibodies” for assay set up).

*Antibody binding sensitivity* is defined as the ratio between the antibody binding signal (as defined above via ELISA) and the total amount of antibody (determined by Bradford assay) used in the reaction for the same preparation (see anti-BMMF Rep antibody binding assay and Bradford methods for the different assay setups).

$$Antibody binding sensitivity= \frac{Antibody binding signal [A450nm-A595nm]}{Antibody protein amount used in this assay [\mu g]}$$

### Competitive anti-BMMF Rep protein binding assay

The specificity of selected antibodies was evaluated with the competitive anti-BMMF Rep antibody binding assay. The basic ELISA setup of this assay was consistent with the anti-BMMF Rep antibody binding assay. However, to test for competition, the selected primary antibody (same concentration as for routine binding assay) was pre-incubated with the respective BMMF Rep protein (or peptide, 50-fold molar excess) that was used for immunization of the antibody (or a mock incubation with the respective elution buffer, as a control) for 1 h at RT in a 100 µl volume. Then, the competition reaction as well as the control reaction were used in parallel in the routine anti-BMMF Rep antibody binding assay described above. The analysis of the same samples was done at least twice. The inhibition of the signal due to competition was calculated in % by comparing antibody reactions with Rep competitor with antibody reactions without Rep competitor relying on the absolute binding signals. A cutoff of ≥ 50% inhibition was used to define antibodies with predominantly competitive and an antigen-specific, displaceable binding.

$$Inhibition (\%)=100- \frac{ Antibody binding signal \left[A450nm-A595nm\right] with competitor \times 100\%}{Antibody binding signal \left[A450nm-A595nm\right] without competitor}$$

### Cross-reactivity of antibodies

The specificity of antibodies to BMMF Rep antigens was further evaluated by testing cross-reactivity of antibodies with selected bacterial lysates and recombinant *E. coli* RepA protein (Cusabio, Houston, TX, USA). Furthermore, mammalian sera, cells and proteins as well as lysate of *E. coli* SoluBL21 bacteria (same strain as used for BMMF Rep production) were analyzed in anti-BMMF Rep antibody binding assay with selected antibodies at least twice in the routine anti-BMMF Rep antibody binding assay. For this, 1 µl of the bacterial lysates (corresponding to 1 × 10^6^ to 5 × 10^6^ colony forming units, [CFU]), mammalian sera from donkey, rabbit, mouse, horse, pig, bovine and human, 200 ng bovine proteins such as casein and BSA and 200 ng of *E. coli* SoluBL21 lysates or human cell lysates were diluted in a final volume of 100 µl PBS and used for coating. Negative controls (background, effects of the sample itself and secondary antibody alone) were assessed. Sample/matrix spiking with defined amounts of BMMF Rep 200 ng/well was performed to rule out assay interference or inhibition.

### Test of false negative effect on samples

To assess a false negative effect (inhibitory effect) of all tested samples on the measured binding signal, samples were additionally spiked with a defined amount of BMMF Rep antigen (200 ng/well) in 100 µl PBS before coating (spiked sample) and assessed in duplicates with the routine anti-BMMF Rep antibody binding assay (except for bacteria lysates, which were tested once). As a control, the same BMMF Rep antigen amount was coated in PBS (spike control) allowing determination of spike recovery in %. PBS and samples without primary antibody were assessed as negative controls. Absolute values were used for calculation of the spike recovery. A cutoff of ≥ 50% spike recovery was defined to identify samples demonstrating a predominantly specific antibody binding.

$$Spike recovery (\%)= \frac{Antibody binding signal \left[A450nm-A595nm\right] of the spiked sample \times 100\%}{ Antibody binding signal \left[A450nm-A595nm\right] of spike control}$$

### Preparation of lysates of bacteria present during milk production

Bacteria for lysate preparations were either isolated from the bulk tank of the LMU research and teaching farm (Lehr- und Versuchsgut [LVG], Oberschleißheim, Germany) or derived from the Chair’s in house strain collection. The bacterial set comprised isolates frequently occurring in close proximity to dairy cows (skin, mammary gland, bedding material, milking devices). The set also contained bacteria that were reported to have a high sequence similarity to the BMMF Rep (*Acinetobacter baumannii*).

For preparation of protein lysates, the isolates were grown in Brain Heart Infusion broth (Oxoid, Thermo Fisher Scientific, Munich, Germany) overnight. Upon harvest, the OD_600_ (Biophotometer (Eppendorf, Hamburg, Germany) was on average around 1.4 ± 0.5, which corresponds to the exponential phase of a bacterial growth curve. Bacteria were pelleted by centrifugation, washed twice in PBS and 1 × 10^9^ to 5 × 10^9^ CFU suspended in one ml lysis buffer (100 mM Tris, 300 mM NaCl,1 mM EDTA, pH 8, containing 0.5 mg/ml lysozyme and protease inhibitor [Complete Mini without EDTA, Roche, Mannheim, Germany]). After ultra-sonication for 2 × 5 min on ice (U200S Control Sonifier, IKA Labortechnik, Staufen, Germany, 100% amplitude and 50% duty cycle), the lysates were cleared by centrifugation 14.000 × g, 20 min at 4 °C. Supernatants were stored at −20 °C and thawed at maximum three times before use. Bacteria lysis buffer and PBS were used as negative controls in ELISA.

### Phylogeny of BMMF Rep antigens with respect to bacterial Reps

Protein sequences of bacterial Rep homologs were identified using BLASTp with the H1MSB.1 Rep sequence as query. Bacterial Rep sequences showing the highest amino acid sequence identity to H1MSB.1 Rep were selected from the NCBI Protein Reference Sequence (RefSeq) database (accessed in Nov. 2025). In total, 13 bacterial Rep sequences were included together with the five BMMF Rep proteins used in this study (Table 2).

**Table 2:**
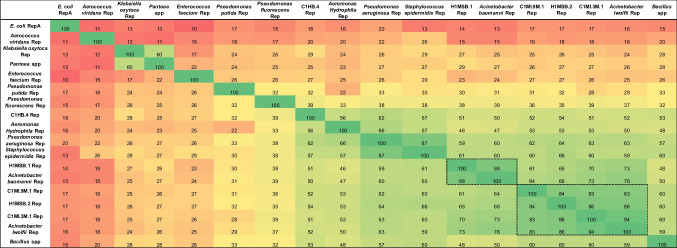
Percentage identity matrix (PIM) generated with Clustal Omega showing the pairwise sequence identities among Rep proteins of BMMF isolates and reference Rep proteins from bacterial species associated with milk production environments

*E. coli* RepA was included as an outgroup. Reference Reps were selected based on the closest homologs to H1MSB.1 Rep identified in the NCBI Protein Reference Sequence (RefSeq) database. The dotted box is showing the bacterial Rep and BMMF Reps with the highest similarity

Multiple sequence alignment was performed using Clustal Omega via EMBL-EBI web interface with default parameters (BLOSUM62 substitution matrix and default gap penalties) including phylogenetic distance-based guide tree (Supplemental Fig. S2, modified with iTOL V 7.5) (Ciccarelli et al. 2006).

### Test of antibody interaction with mammalian-associated sera, cells and protein

To further test specificity of the BMMF antibodies, we assessed antibody reactivity with mammalian specimens, including fetal bovine sera (Sigma Aldrich, St. Louis, MO, USA), casein from bovine milk (Sigma Aldrich, St. Louis, MO, USA), bovine serum albumin (Thermo Fisher Scientific, Waltham, MA, USA), as well as sera from human, donkey (Biotrend, Cologne, Germany), rabbit, mouse, horse, and pig (Pan-Biotech, Aidenbach, Germany) and human embryonic kidney (HEK) cell lysate (ATCC Manassas, VA, USA, CRL-11268, clone 17) (in duplicates). For this, one µl of different mammalian sera, 200 ng bovine proteins such as casein and BSA and 200 ng of *E. coli* SoluBL21 lysates or human cell lysates were diluted in 100 µl PBS per well, coated and assessed in the routine anti-BMMF Rep antibody binding assay (for controls and assay set up see chapter “Anti-BMMF Rep antibody binding assay”).

## Results

Antibodies reactive against the conserved Rep protein of BMMF1 isolates have represented cornerstones in observing increased histologic expression of BMMFs in cancer patients and thus in analyzing BMMFs’ role as potential biomarkers for cancer and potential diagnostic applications. Consequently, careful and systematic description of their biochemical qualities and detection characteristics against BMMF and potential non-BMMF Rep antigens is of utmost importance. Therefore, in this study, we expanded previous analyses on mAb1-11, 13–15, for which data on BMMF antigen reactivity and epitope detection as well as histological BMMF Rep detection in patient tissues partially exist already (Bund et al. 2021; Nikitina et al. 2024; Siqin et al. 2025), and included four new antibodies directed against new BMMF Rep targets (mAb20 and mAb21: directed against C1HB.4 Rep, mAb22 and mAb23: H1MSB.2, C1MI.3M.1, C1MI.9M.1 Rep) of isolates observed in the cancer context in genomic analyses (Häfele 2024). Finally, we characterized 18 individual anti-BMMF Rep mouse antibodies as well as one recombinant human and one recombinant rabbit version of mAb3, the most frequently applied antibody in clinical histological analyses, in one combined, comprehensive analysis, regarding epitope mapping, appearance, subtype, protein- and IgG- content, as well as relative size distribution of the antibody preparations. We assessed antibody reactivity, the specificity and binding sensitivity based on five different BMMF Rep protein antigens, three distinct subdomains as well as three peptides. Additionally, for 15 selected anti-BMMF Rep antibodies, we evaluated possible cross-reactivity of the Rep antibodies to bacterial lysates, *E. coli* SoluBL21 lysate, mammalian sera, cells, and protein samples of different eukaryotic origin.

### Development and production of anti-BMMF Rep antibodies

The anti-BMMF Rep monoclonal mouse antibodies (mAb) 1–11 and 13–15 were generated and characterized before (Bund et al. 2021). Antibodies 20 and 21 were generated newly by immunization with C1HB.4 full-length Rep antigen and mAb22 and mAb23 via triple immunization with the Reps of H1MSB.2, C1MI.3M.1 and C1MI.9M.1. New human (hAb3) and rabbit (rAb3) recombinant chimeric versions of the mouse antibody mAb3 were produced upon recombinant expression of the identified variable regions of mAb3 together with the respective human and rabbit constant heavy (CH) and kappa light (CL) chains (Wardemann and Busse 2019). Generally, all purified antibodies were formulated in PBS buffer at −80 °C for long-term and at 4 °C for short-term storage. Storage at −80 °C ensured long-term stability of the test throughout the period of approximately 12 months used for data collection and by application of the routine anti-BMMF Rep binding assay with defined Abs and Reps.

### Characterization of produced BMMF Rep proteins

To evaluate the antigen reactivity of the antibodies, five BMMF1 Rep proteins, including H1MSB.1, H1MSB.2, C1HB.4, C1MI.3M.1, and C1MI.9M.1 Rep plus the H1MSB.1 Rep-respective subdomains Winged-helix WH1 (aa 1–136), WH1 + WH2 (aa 1 to aa 229), WH2 + C-terminus (WH2 + C-term, aa 137 to aa 324), were produced, purified and formulated in both NaCa and PBS buffers (Table 1). Peptides 1 and 2 were produced for reactivity analysis and represent consensus peptide sequences contained in the Reps of several BMMF1 isolates. Peptide 3 was synthesized to confirm the proposed linear epitope sequence of mAb22 and mAb23.

### Characterization of anti-BMMF Rep antibodies

We first assessed basic characteristics of the antibodies, such as biophysical appearance (no turbidity observed), subtype, protein-, IgG- quantification and relative size distribution, and mapped the epitopes for individual Rep antibodies (Table 3).

**Table 3:** Characteristics of 20 anti-BMMF Rep antibodies including subtype, immunization Rep antigen, epitope sequence and position, antibody protein concentration (measured via Bradford assay) and IgG concentration (measured by immunoglobulin ELISA) as well as purity in % (ratio of IgG to protein content)

Antibody	Subtype	Immunization Rep antigen	Epitope sequence	Epitope position	Protein concentration mg/ml	IgG concentration mg/ml	Purity %	Antibody binding sensitivity
mAb1	IgG2a	Peptide 2	EHTDITASY	200–208	0.10	0.27	100	+ + + +
mAb2	IgG2a	Peptide 1	DPLTVH	44–49	0.15	0.09	60	+ + + +
mAb3	IgG1	H1MSB.1	WESKLEEFGVV	313–323	1.20	1.71	100	+ + +
hAb3	IgG1				0.87	2.83	100	+ + + + +
rAb3	IgG1				0.14	n.a	n.a	+ + + + +
mAb4	IgG2b	H1MSB.1	DPLTVH, EHTDITASY	44–49, 200–208	0.18	0.21	100	+ + +
mAb5	IgG2b	Peptide 1	DPLTVH	44–49	0.12	0.17	100	+ + +
mAb6	IgG2b	H1MSB.1	QINEHTDITASYE	197–209	0.10	0.12	100	+ + +
mAb7	IgG1		QINEHTDITASYE	197–209	0.07	0.10	100	+ + +
mAb8	IgG1		WH1 + C-term	inconclusive	0.44	0.29	70	+ + +
mAb9	IgG1		NRLSDRF	280–286	0.09	0.23	100	+ + + +
mAb10	IgG1		WESKLEEFGVV	313–323	1.44	1.83	100	+ +
mAb11	IgG2b		WH1 + C-term	inconclusive	0.23	0.20	90	+ + + +
mAb13	IgG2a		WH1	inconclusive	0.10	0.16	100	+ + + +
mAb14	IgG2b	Peptide 1	ANDPLTVH	42–49	0.37	0.23	60	+ +
mAb15	IgG2b		LTVH	46–49	0.67	1.32	100	+ +
mAb20	IgG2c	C1HB.4	inconclusive	inconclusive	0.53	0.17	30	+
mAb21	IgG2b		inconclusive	inconclusive	0.11	0.30	100	+ + + +
mAb22	IgG2c	H1MSB.2, C1MI.3M.1 and C1MI.9M.1	LKTETDYSKKN	309–319, 307–318, 311–322	0.37	0.62	100	+ + + +
mAb23	IgG2c		LKTETDYSKKN		0.64	1.75	100	+

The antibody binding sensitivity (ratio between the antibody binding signal and the antibody protein amount used in this assay [µg]) is determined for individual reactions of H1MSB.1 Rep together with mAb1-11, 13–15, C1HB.4 Rep with mAb20 and mAb21 and H1MSB.2 Rep with mAb22 and mAb23. Classification of antibody binding sensitivity: A_450nm_-A_595nm_/µg antibody (protein) ≤ 0.2: +; > 0.2 ≤ 0.5: + +; > 0.5 ≤ 1: + + +; > 1 < 100: + + + + and ≥ 100: + + + + +

**Fig. 1:**
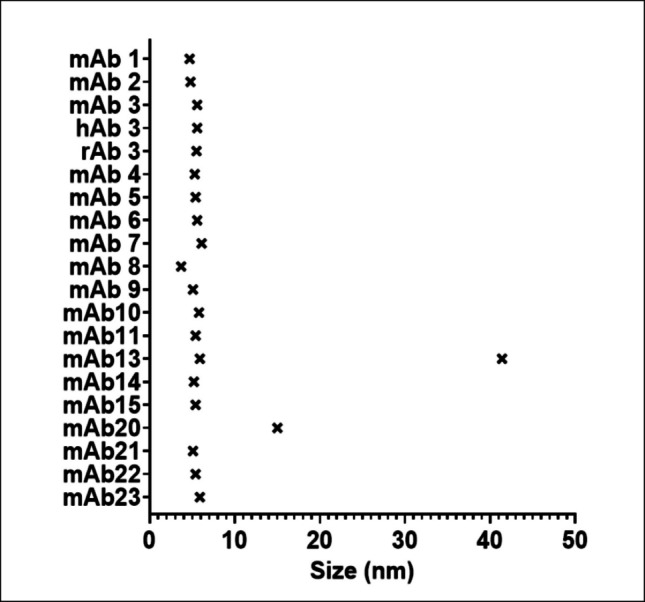
Distribution of hydrodynamic diameters measured for the anti-BMMF Rep antibody preparations via dynamic light scattering. Representation of the size (in nm) of the peak population of molecules in the solution. Two peak populations were identified for mAb13. Each value represents a mean of 10 single measurements of one sample

All antibodies were classified as immunoglobulin class G antibodies. The anti-BMMF Rep mAb3, 7, 8, 9, 10 were marked as IgG1 subclass. MAb1, 2, 13 were identified as IgG2a, whereas mAb4, 5, 6, 11, 14, 15, 21 were designated as IgG2b subclass and mAb20, 22, 23 were assigned to IgG2c subclass. Further, we assessed antibody purity, defined as the ratio of IgG concentration (IgG ELISA) to the protein concentration (Bradford) of a given antibody preparation. All tested antibodies showed a high purity of more than 90%, except for mAb2, 8, 14 with a purity between 60–70%. MAb20 demonstrated a 30% purity. Additionally, we evaluated the quality of the antibodies by size distribution via Dynamic Light Scattering (DLS). All antibodies demonstrated a main hydrodynamic radius peak of 5.4 ± 0.5 nm with a relative frequency of 13 ± 4%. MAb13 showed an additional peak of 41 nm, albeit with a low relative frequency of 4%. MAb20 exhibited a main peak shifted to a larger size of 15 nm with a relative frequency of 10% (Fig. 1).

### Development of anti-BMMF Rep antibody binding assay for analysis of antibody reactivity, specificity, binding sensitivity and unspecific cross-reactivity

The ELISA-based anti-BMMF Rep antibody binding assay was developed to determine the reactivity of the antibodies to the different BMMF Rep antigens or Reps that might endogenously be present in other test samples. The antibody binding signal observed in negative controls without Rep was below 0.1, demonstrating no Ab interaction with the corresponding matrix (background)._._ The cutoff for antibody reactivity was defined with a binding signal of ≥ 0.3. For determination of assay accuracy, the combination of H1MSB.1 Rep antigen with mAb3 detection was used in all assays as a reference. In 33 individual measurements, this combination revealed a coefficient of variation (CV) of approximately 10% (Supplemental Fig. S1, A) that was within the acceptable CV of 15% as defined by the European Medicines Agency (EMA 2022).

#### Reactivity of anti-BMMF Rep antibodies against Rep antigens

Reactivity of anti-BMMF Rep antibodies was tested against full length Reps, Rep domains and peptides either in NaCa or PBS buffer formulation (Table 4, Supplemental Table S1, reproduced at least twice). In total, all analyzed antibodies reacted against at least one BMMF antigen, supported by an antibody binding signal of ≥ 0.3. 69 (31%) of all individual anti-BMMF Rep antibody-antigen combinations resulted in positive reactions, while 151 (69%) combinations were considered negative with an antibody binding signal of < 0.3. The anti-penta His antibody confirmed appropriate detection of all His-tagged antigens except for the *E. coli* RepA (no His tag), which was detected exclusively with the commercial anti-RepA antibody. None of the anti-BMMF Rep antibodies showed any binding to the commercially available *E. coli* RepA protein. Mouse IgG1 isotype control antibody demonstrated no reactivity with any of the tested antigens.

**Table 4:**
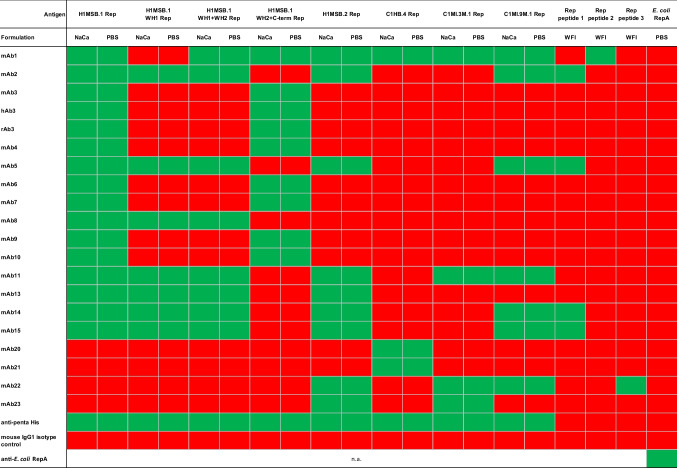
Binding reactivity of anti-BMMF Rep antibodies to BMMF Rep proteins and peptides formulated either in sodium-carbonate buffer (NaCa), phosphate buffer (PBS) or water for injection (WFI)

An antibody is defined as reactive if the antibody-specific binding signal exceeds 0.3 (A_450nm_-A_595nm_) (green, equaling at least 3 × the anti-BMMF Rep antibody binding assay background intensity). Antibody binding signals below 0.3 were considered negative (red)

Comparison of data generated with antigens formulated in NaCa and PBS (Supplemental Table S1) showed an almost perfect correlation (*p* < 0.0001, *r* = 0.99 [Pearson]), confirming robustness of the detection, and indicated similar binding independent of the two buffer conditions.

For a more detailed characterization of antibody reactivity against the different Rep antigens, antibodies were grouped according to the different immunization strategies used for the antibody production (Table 5).

**Table 5:**
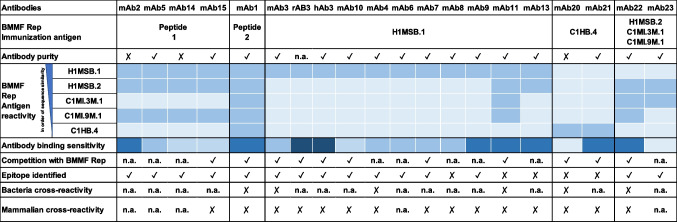
Summary of the characteristics of anti-BMMF Rep antibodies, grouped by immunization

The antibody purity is defined as the ratio of IgG to protein concentration. A purity of 70% and above is visualized with ✔ and below 70% with ✘. The reactivity is defined as the ability of an antibody to bind anti-BMMF Rep proteins or peptides with an antibody-specific absorption (binding signal) ≥ 0.3 (A_450nm_-A_595nm_). Antigen reactivity is considered negative for binding signals < 0.3 (light blue) and positive ≥ 0.3 (darker blue). Antibody binding sensitivity is defined as the ratio between the antibody binding signal (A_450nm_-A_595nm_) per µg of antibody used (with H1MSB.1 Rep antigen used for reactions with mAb1-11, 13–15, C1HB.4 Rep for mAb20 and 21 and H1MSB.2 Rep for mAb22 and mAb23). Antibody binding sensitivity is classified as follows highlighted by a blue color gradient: ≤ 0.2: +; > 0.2 ≤ 0.5: + +; > 0.5 ≤ 1: + + +; > 1 < 100: + + + + and ≥ 100: + + + + + (darkest shade of blue). Competition of the anti-BMMF antibody binding to the respective immobilized antigen was tested based on antibody-antigen pre-incubation. A competition resulting in ≥ 50% inhibition when compared to the reaction without antigen pre-incubation is depicted with ✔ (< 50% with ✘). Epitope positions predicted via peptide epitope mapping which were confirmed via the antibody binding assay are depicted with ✔ (otherwise ✘). Reactivity of BMMF antibodies with bacterial lysates or mammalian specimens was tested via the binding assay; no reactivity: ✘, reactivity: ✔ (not observed), not analyzed: n.a.

Consensus antibodies mAb2, 5, 14, 15 (peptide 1 immunization) showed binding to H1MSB.1, H1MSB.1 WH1- and WH1 + WH2 fragment, H1MSB.2, C1MI.9M.1 Rep protein and peptide 1 (as expected). MAb1 (peptide 2 immunization) demonstrated binding to an even larger spectrum of antigens including H1MSB.1, H1MSB.1 WH1 + WH2 fragment, H1MSB.1 WH2 + C-terminus fragment, H1MSB.2, C1HB.4, C1MI.3M.1, C1MI.9M.1 Rep protein and peptide 2 (as expected). These findings confirmed and expanded our previous observation (Bund et al. 2021) that consensus antibodies originating from immunization with peptides derived from conserved Rep amino acid stretches are able to react with several Rep antigens of isolates from the BMMF1 subgroup.

In contrast, all but two of the antibodies originating from H1MSB.1 Rep-specific immunization reacted only against the Rep of H1MSB.1, or fragments thereof. This included also the rabbit (rAb3) and human (hAB3) recombinant chimeric versions of mAb3. Besides against H1MSB.1 Rep, mAb11 was also reactive against the Reps of H1MSB.2, C1MI.3M.1 and C1MI.9M.1, while mAb13 was additionally reactive against H1MSB.2 Rep.

With regard to the detected Rep subdomains of H1MSB.1, mAb3, 4, 6, 7, 9, 10 reacted with WH2 + C-terminus fragment, while mAb2, 5, 8, 11, 13–15 reacted with the H1MSB.1 Rep WH1 fragment and the WH1 + WH2 fragment, and mAb1 with WH1 + WH2 fragment and WH2 + C-terminus fragment (Table 4).

The exclusively C1HB.4 Rep-immunized mAb20 and mAb21 only reacted with the C1HB.4 Rep, indicating high target specificity.

MAb22 and mAb23 raised upon triple immunization with the Reps of H1MSB.2, C1MI.3M.1 and C1MI.9M.1 exclusively reacted with antigens of this group. While mAb22 reacted with all three antigens, mAb23 only showed reactivity against the Reps of H1MSB.2 and C1MI.3M.1.

#### Anti-BMMF Rep antibody binding sensitivity

Anti-BMMF Rep antibody binding sensitivity was assessed in a semi-empirical way and defined as the ratio between the antibody binding signal and the amount (µg) of antibody used (antibody protein amount determined by Bradford assay). For mAb1-11, 13–15, hAb3 and rAb3, the binding sensitivity was tested with H1MSB.1 Rep, for mAb20 and 21 with C1HB.4 Rep, and for mAb22 and mAb23 with H1MSB.2 Rep antigens (based on the immunization strategy). MAb1, 2, 3, 4, 5, 6, 7, 8, 9, 11, 13, 21 and 22 demonstrated a high antibody binding sensitivity of > 0.5 (considered + + +, + + + + or + + + + +) (Table 3). MAb10, 14 and 15 revealed intermediate antibody binding sensitivity between 0.3 and 0.5 (+ +). MAb20 and mAb23 demonstrated a low antibody binding sensitivity of 0.1 (+).

Taken together, the 20 different anti-BMMF Rep antibodies showed reactivity against specific full-length BMMF1 Rep proteins and H1MSB.1 Rep domains, with 15 antibodies (75%) showing high, three antibodies (15%) showing intermediate and two antibodies (10%) showing low binding sensitivity. The recombinant versions rAb3 and hAb3 revealed an around one log higher binding sensitivity compared to mAb3.

### BMMF Rep antibody specificity

#### No cross-reactivity against bacterial lysates associated with milk production

To find out whether the produced anti-BMMF Rep antibodies are specifically reactive against BMMF Reps or whether they would also react with the Reps of bacteria, which occur e. g. during milk production, we evaluated the cross-reactivity of selected antibodies (mAb1, 3, 4, 11, 20, 22) with a total of 21 bacterial lysates stemming from 16 different bacterial strains (Supplemental Table S2) including *Acinetobacter baumannii*, *Acinetobacter junii/lwoffii*, *Acinetobacter lwoffii*, *Aerococcus viridans*, *Aeromonas hydrophila*, *Bacillus* spp*, Brevundimonas vesicularis*, *Enterococcus faecium*, *Klebsiella oxytoca*, *Pantoea* spp, *Pseudomonas aeruginosa*, *Pseudomonas fluorescens*, *Pseudomonas putida*, *Sphingomonas paucimobilis*, *Staphylococcus chromogenes* and *Staphylococcus epidermidis*.

The antibodies used in this assay covered mAb1, as the peptide-based antibody with the broadest range of reactivity to different Reps observed, so far (5/5 different Reps detected), mAb3, as H1MSB.1-Rep selective and most frequently applied antibody in clinical histology, mAb11, as H1MSB.1-immunized antibody but with a different reaction profile also including other Reps (and positive in CRC IHC), mAb20 for detection of C1HB.4 Rep and mAb22 for detection of C1MI.9M.1, H1MSB.2 and C1MI.3M.1 Reps. Additionally, mAb4 was included with the same binding profile as mAb3, however with different (so far inconclusive) epitope sequence.

Neither of the tested antibodies showed any cross-reactivity with the collection of analyzed bacterial lysates (Supplemental Table S2). Even lysates of bacteria that might harbor potential Reps with a high sequence similarity to Reps of H1MSB.1, C1MI.9M.1, H1MSB.2 and C1MI.3M.1 (Table 2), determined via protein BLAST (highlighted in a dotted box) did not show a reaction, suggesting either absence of the antigen or lack of detectable binding to the respective BMMF antibodies under the applied assay conditions.

To demonstrate that the bacterial sample matrix does not cause inhibition of the antigen–antibody interaction (false negative effect), defined amounts of purified BMMF Rep proteins were used as positive control antigens and were spiked into the lysates before coating and compared with BMMF antigen coating in PBS. All tested antibodies demonstrated no inhibitory effect with recoveries higher than 50% (Supplemental Table S3). The lysis buffer itself containing either lysozyme or protease inhibitor showed no inhibition of detection of the BMMF Rep protein with a recovery of 97 ± 4% with the corresponding antibodies when applied in the routine test setup. Of note, without Rep coating, all tested conditions with lysis buffer (undiluted, diluted 1:10 and diluted 1:100), alone, resulted in no notable binding assay signal (< 0.1) for all antibodies used.

#### Test of BMMF antibody cross-reactivity with mammalian specimens

To assess a potential use of the anti-BMMF Rep antibody panel in a serological context, we also tested for potential cross-reactivity to sera, cells and proteins of different mammalian sources. No cross-reactivity was observed with mammalian specimens derived from bovine, human, donkey, rabbit, mouse, horse and pig sera, human cells, bovine proteins as well as the *E. coli* SoluBL21 clone used for the BMMF Rep production (Table 6). A potential cross-reaction of the humanized chimeric antibody (hAb3) with human, rabbit and pig sera was observed (e. g. interaction of rAb3 with rabbit serum), but obviously caused by the cross-interaction with the respective secondary HRP detection antibody (which was demonstrated by’no primary Ab’ detection controls for these sera). Mouse sera exclusively interacted with the mouse antibodies caused by the binding of the secondary anti-mouse HRP detection antibody to the mouse sera. Rabbit sera demonstrated the same result with the rAb3 caused by interaction of the anti-rabbit HRP detection antibody with the rabbit sera. A mean spike recovery of 65% for mAb1, 3 and 22 demonstrated negligible to mild inhibitory effects for the tested sera under the given dilution (1:100) (Supplemental Table S3).

**Table 6:** Cross reactivity of anti-BMMF Rep antibodies with different mammalian specimens (bovine, human, donkey, rabbit, mouse, horse and pig samples) as well as host cell lysate of *E. coli* SoluBL21, which was used for BMMF Rep protein production

		**Antibody**							
**Source**		**mAb1**	**hAb3**	**mAb3**	**rAb3**	**mAb4**	**mAb7**	**mAb8**	**mAb9**
**Bovine**	Fetal sera	-	-	-	-	-	-	-	-
	Casein	-	-	-	-	-	-	-	-
	Serum albumin	-	-	-	-	-	-	-	-
**Human**	HEK cell lysate	-	-	-	-	-	-	-	-
	Human sera	-	+ +	-	-	-	-	-	-
**Diverse mammalian**	Donkey sera	-	-	-	-	-	-	-	-
	Rabbit sera	-	+ +	-	+ +	-	-	-	-
	Mouse sera	+ +	-	+ +	-	+ +	+ +	+ +	+ +
	Horse sera	-	-	-	-	-	-	-	-
	Pig sera	-	+ +	-	-	-	-	-	-
***E. coli* SoluBL21**	Cell lysate	-	-	-	-	-	-	-	-

**Source**	**mAb10**	**mAb11**	**mAb13**	**mAb15**	**mAb20**	**mAb21**	**mAb22**	**NC** **no primary Ab**	**NC** **only sample**
**Bovine**	-	-	-	-	-	-	-	-	-
	-	-	-	-	-	-	-	-	-
	-	-	-	-	-	-	-	-	-
**Human**	-	-	-	-	-	-	-	-	-
	-	-	-	-	-	-	-	+ +	-
**Diverse mammalian**	-	-	-	-	-	-	-	-	-
	-	-	-	-	-	-	-	+ +	-
	+ +	+ +	+ +	+ +	+ +	+ +	+ +	+ +	-
	-	-	-	-	-	-	-	-	-
	-	-	-	-	-	-	-	+ +	-
***E. coli* SoluBL21**	-	-	-	-	-	-	-	-	-

Cross reactivity is defined as antibody binding signal (A_450nm_-A_595nm_) and classified as follows: ≤ 0.3: -; > 0.3 ≤ 1: +; > 1: + +. “NC no primary Ab” evaluates a sample with only secondary Ab while “NC only sample” is a sample without primary and secondary Abs

We also evaluated spike recovery in mammalian specimens (Supplemental Table S3). For mAb1, the spike recovery (tested in duplicates) for all tested sera samples was above 50%, except for mouse serum, which showed a recovery of only 15%. MAb3 revealed a spike recovery between 30 and 78%, except for mouse sera with around 20%. The spike recovery with the mAb20 was weak (lower than 30%) for all analytes, except for bovine casein and sera albumin with more than 50%. With the mAb22, the spike recovery was above 75%, except for mouse sera with around 40%.

#### No cross-reactivity of anti-BMMF Rep antibodies to the E. coli SoluBL21

In addition, no cross-reactivity of anti-BMMF Rep antibodies with the *E. coli* SoluBL21 lysate used for BMMF Rep protein production was observed (Table 6). The spike recovery with the selected BMMF antibodies mAb1, 3 and 22 exceeded 87%, demonstrating no inhibitory effect on the assay (Supplemental Table S3). MAb20 showed a spike recovery of only 36%.

#### Specificity confirmation of selected anti-BMMF Rep antibodies via competition assay

To confirm the antibody specificity, a competitive anti-BMMF Rep antibody binding assay was performed based on representative antibodies of the different immunization groups (mAb1, 3, 7 10, 11, 15, 20, 21, 22, hAb3 and rAb3) and their respective BMMF Rep antigens. In this setup, a pre-incubation of the antibody with respective antigens (50-fold molar excess) was used to compete with the antibody binding to Rep antigens immobilized to the plate (the latter as the usual setup). All tested antibodies demonstrated specificity with a signal inhibition exceeding 50% (Table 7). The following inhibition rates were observed for mAb22: with C1MI.9M.1 Rep: 63%, H1MSB.2 Rep: 95%, H1MSB.2 peptide: 92%. However, the combination of mAb22 with C1MI.3M.1 Rep antigen was able to achieve over 50% signal inhibition only with a 250-fold molar excess (83%). Competition with peptide 2 for the mAb1 and with peptide 1 for the mAb15 also yielded 50% signal inhibition only with a 250-fold molar excess (56% and 68%, respectively).

**Table 7:** Specificity of anti-BMMF Rep antibodies shown via the competition of anti-BMMF Rep antibody binding signal

Conditions				Immunogen inhibition %	
	**Rep Immunogen**	**Rep** **Ag coating** **with**	**Rep** **Ag competition** **with**	**50-fold molar Ag excess to Ab**	**50-fold molar hCP excess to Ab**
**mAb1**	Peptide 2	H1MSB.1	Peptide 2* H1MSB.2	17/56^#^ 69	4
**hAb3**	H1MSB.1	H1MSB.1	H1MSB.1	52	0
**mAb3**	H1MSB.1	H1MSB.1	H1MSB.1	84	4
**rAb3**	H1MSB.1	H1MSB.1	H1MSB.1	76	11
**mAb7**	H1MSB.1	H1MSB.1	H1MSB.1	68	0
**mAb10**	H1MSB.1	H1MSB.1	H1MSB.1	96	5
**mAb11**	H1MSB.1	H1MSB.1	H1MSB.1	74	0
**mAb15**	Peptide 1	H1MSB.1	H1MSB.2 Peptide 1*	93 23/68^#^	11
**mAb20**	C1HB.4	C1HB.4	C1HB.4	91	90
**mAb21**	C1HB.4	C1HB.4	C1HB.4	84	0
**mAb22**	H1MSB.2 C1MI.3M.1 C1MI.9M.1	H1MSB.2	H1MSB.2	95	0
			C1MI.3M.1	41/83^#^	
			C1MI.9M.1	63	
		Peptide 3	Peptide 3*	92	

The competition (in %) is calculated by comparing the antibody binding signal (A_450nm_-A_595nm_) with competitor (i.e. including antigen–antibody pre-incubation) to the antibody binding signal without competitor (no pre-incubation of the antibody with antigen). The BMMF Rep antigens used for both coating and pre-incubation were denatured, while the peptides used for competition were applied in their native form (*). Peptide 3 was coated under native conditions. A 50-fold molar antigen excess to antibody was used for the pre-incubations except for three reactions, where a 250-fold molar excess of antigen was utilized (^#^). Abbreviations—antibody: Ab, antigen: Ag, host cell protein: hCP

We further proved the specificity of anti-BMMF Rep antibodies by using *E. coli* SoluBL21 lysate as a competitor for these selected antibody antigen combinations. None of the combinations suffered any inhibition when tested with prokaryotic lysate except for mAb20, which demonstrated 90% inhibition.

In summary, these findings confirmed that the anti-BMMF Rep antibodies are suitable for targeted detection of different BMMF Rep proteins in strong concordance with the antibody immunization used for the given antibody. Furthermore, our results demonstrated that the antibodies did not show any detectable cross-reactivity against the tested bacterial lysates, mammalian specimens and *E. coli* SoluBL21 host cell proteins, thus demonstrating specificity.

## Discussion

Antibodies represent indispensable tools for biomarker research and diagnostics. Application of antibodies in immunohistochemistry and spatial proteomics enables the localization and quantification of disease-associated protein targets in specific tissues. Such approaches complement genomic and transcriptomic data by validating phenotypical changes at the protein level. In this context, antibodies directed against the conserved replication protein (Rep) of Bovine Meat and Milk Factors (BMMFs) have been pivotal for demonstrating BMMF Rep expression in colorectal and other cancers, supporting BMMFs’ proposed role as potential biomarkers or even infectious cofactors in carcinogenesis (Bund et al. 2021; Nikitina et al. 2024). Earlier work by Yeh et al. (2017) and colleagues highlighted the utility of polyclonal, peptide-generated antibodies recognizing Sphinx/Rep elements in mammalian tissues, and this effort has since expanded to include several monoclonal antibodies targeting expression of BMMF Rep proteins frequently detected in cancer lesions (Bund et al. 2021). However, the available BMMF antibody repertoire has not been comprehensively characterized regarding its reactivity and specificity against an extended range of possible BMMF and non-BMMF Rep antigens. In this study, we established an ELISA-based anti-BMMF Rep antibody binding platform to benchmark monoclonal anti-BMMF Rep antibodies for reactivity, specificity, and binding sensitivity and further evaluated purity and size distribution.

Except for mAb2, 8, 14 and 20, all antibodies exhibited a purity of more than 90% (Table 5). This is in line with the commercial anti-penta His and anti-*E. coli* RepA purity (> 90%). Importantly, the purity and subclass of the different antibody preparations did not correlate with the antibody binding sensitivity, except for mAb14 and mAb20. The binding sensitivity might be negatively influenced by the reduced purity for mAb14 and mAb20. Importantly, however, the antigen specificity profile of mAb20 remained restricted exclusively to C1HB.4 Rep. Furthermore, the low spike recovery in combination with the inhibition by *E. coli* SoluBL21 lysates as competitor might indicate that respective mAb is prone to matrix effects, although no cross-reactivity with other BMMF Reps, bacterial lysates, or mammalian samples was observed. Dynamic light scattering analysis confirmed that most antibodies were monodisperse, with a dominant hydrodynamic diameter of approximately 5.4 nm, consistent with the size of native IgG molecules (Gagnon et al. 2015). The single, narrow peak observed for most samples indicates that the purified preparations maintained structural integrity and lacked detectable aggregation. In contrast, mAb13 displayed a minor secondary peak at ~ 41 nm, corresponding to a small fraction of higher-order aggregates. This behavior likely reflects transient conformational stress introduced during protein A based purification, a phenomenon known to affect certain antibody clones (Gagnon et al. 2015). Similarly, mAb20 exhibited a broader size distribution with a primary peak near 15 nm, suggesting partial oligomerization. Such deviations from monodispersity highlight intrinsic structural differences among antibody clones and emphasize the need for batch-specific quality control. To enhance purity and deplete aggregates, the up- and downstream antibody production process should be standardized and the quality could be improved by further purification steps like ion exchange or size exclusion chromatography if more demanding downstream applications are envisioned (Jafarzadeh Chehraghi et al. 2025).

Taken together, out of the 20 tested anti-BMMF Rep antibody batches, only mAb2, 8, 14, and 20 revealed inconsistencies regarding either purity and/or dispersity, which might be ameliorated in the production process before future application.

We next subjected the 14 previously (mAb1-11, 13–15) and six newly generated antibodies (mAb20-23, rAb3, hAb3) to a reactivity test against BMMF Rep antigens with the qualified anti-BMMF Rep antibody binding assay. This assay was qualified demonstrating dilutional linearity, determining assay range, demonstrating accuracy of 10% CV for quality control reactions and ensuring sufficient recovery with guaranteed antibody stability at −80 °C. Overall, the ELISA based platform was evaluated as robust while showing reproducible data with confirmed quality controls. The tested panel of antigens for the first time included also BMMF Rep antigens associated with newly identified, clinically resolved BMMF1 isolates, such as H1MSB.2, C1HB.4, C1MI.3M.1 and C1MI.9M.1 Rep (Siqin et al. 2025).

When grouping the panel of tested antibodies according to their immunization strategy used for antibody production, antibodies raised against the two consensus Rep peptides showed an expected broad reactivity to BMMF1 Reps (Table 5). MAb1 generated against the consensus Rep peptide 2 (KQINEHTDITASYEQHKKGRT) bound H1MSB.1, H1MSB.2, C1HB.4, C1MI.3M.1 and C1MI.9M.1 encoded Rep protein, which is in line with the presence of the antibody epitope core sequence (EHTDITASY) (Bund et al. 2021). Although there is an amino acid substitution present in the epitope core sequence between the Reps of some of the isolates, the changes are isofunctional (alanine to valine) or are localized at the extreme C-terminus end of the sequence (serine to threonine), leading to no effect on the reactivity profile of this antibody. In contrast, mAb2, 5, 14 and 15 raised against the consensus Rep peptide 1 (EARETGKGINANDPLTVH) detected H1MSB.1, H1MSB.2 and C1MI.9M.1 encoded Rep protein, but did not detect C1HB.4 and C1MI.3M.1 Rep. This is explainable by the presence of the core epitope sequence (ANDPLTVH) (Bund et al. 2021) in the Reps of H1MSB.1, H1MSB.2 and C1MI.9M.1. Concerning lack of detection with C1MI.3M.1 Rep, although the core epitope sequence is fully present in C1MI.3M.1 Rep, the flanking sequence showed a single amino acid change at position 34 from isoleucine to leucine, indicating that side chain interactions or other structural effects might affect the reactivity. In C1HB.4 Rep, the epitope core sequence was absent, explaining lack of reaction. These findings of antibody interaction align with Bund et al. (2021), where mAb1, 2, 5, 11, 14 and 15 detected the Reps of H1MSB.1, H1MSB.2 and C1MI.1.

Almost all antibodies raised against full-length H1MSB.1 Rep (mAb3, 4, 6, 7, 8, 9 and 10) bound selectively to H1MSB.1 Rep, consistent with the immunization strategy and confirming antigen-specific responses. In contrast, mAb11 additionally recognized the Reps of H1MSB.2, C1MI.3M.1, and C1MI.9M.1, while mAb13 detected both H1MSB.1 and H1MSB.2 Rep antigens. Bund et al. (2021) showed that antibody epitopes of several antibodies in this group could not be mapped to linear epitopes, but instead were localized to larger Rep domains using WB and immunofluorescence analyses of H1MSB.1 Rep subdomain constructs. Specifically, mAb8 and mAb11 and their epitopes, respectively, were previously assigned to the WH1 + C-terminal region, while the epitope of mAb13 was localized to WH1. Our ELISA data supported reactivity to the WH1 and WH1 + WH2 fragments for mAb8, 11 and 13, however not to the C- terminal fragment for mAb8 and 11. Taken together, the ELISA data allowed to more accurately map a potential epitope region to the Rep WH1 for mAb8, 11, and 13, however without identification of a clear linear epitope. The observation that mAb11 and mAb13 also detect Reps other than H1MSB.1 Rep used for immunization might suggest the presence of a conserved, structural epitope in WH1 which is recognized by those two antibodies. It is important to note that the Rep WH1 region of structurally similar Reps of *Pseudomonas syringae* RepA generally forms well-defined, tertiary structures (including a conserved winged-helix, “WH” fold) and WH1 was shown to be crucial for dynamic structural processes involving Rep dimerization, but also oligomerization and potential cytotoxic filamentation (Kilic et al. 2019; Torreira et al. 2015). The underlying tertiary or quaternary structures or parts thereof might be shared among different Reps which might explain the broad interaction profile of mAb11 (H1MSB.1 Rep, plus H1MSB.2, C1MI.3M.1, and C1MI.9M.1 Rep) and mAb13 (H1MSB.1 Rep, plus H1MSB.2), while mAb8 seems to identify H1MSB.1 Rep structures, more specifically (no interaction with Reps of other isolates). Importantly, for the lead antibody mAb3 and the new recombinant versions rAb3 and hAb3, selective interaction with the H1MSB.1 full-length Rep and only the WH2 + C-terminal Rep domain was detected, which consistently supports the description of a C-terminal epitope by previous studies (Bund et al. 2021), including the isofunctional mAb10.

The new antibodies raised against the full-length C1HB.4 Rep (mAb20 and mAb21) were highly specific and exclusively reacted with the C1HB.4 Rep, with no reactivity with other BMMF1 isolates or consensus peptides. Unfortunately, epitope mappings performed in the current study did not identify any consistent linear epitopes, suggesting involvement of 3D structure epitopes in antibody antigen interaction. The lack of interaction of this antibody with other Rep antigens is explainable by the large sequence variation of the C1HB.4 Rep when compared to any other BMMF1 Rep (max 54% sequence similarity to the Rep of H1MSB.2 as closest BMMF Rep).

Further new antibodies, mAb22 and mAb23, which were raised against a cluster of three Rep antigens (encoded by H1MSB.2, C1MI.3M.1, and C1MI.9M.1) detected the expected antigens in the binding assay. While mAb22 detected all three BMMF antigens, mAb23 only detected the Reps of H1MSB.2 and C1MI.3M.1. The reason for this might be that although the proposed linear epitope of these antibodies (LKTETDYSKKN, confirmed via Rep peptide 3) is present in all three antigens, a single amino acid substitution in the upstream flanking region of C1MI.9M.1 Rep (at position 304, isoleucine to alanine) or other side chain order structural effects might slightly affect the reactivity in this rather physiologic test for molecular interaction. In general, the observation that a triple immunization with three distinct full-length Rep antigens resulted in generation of two strong antibodies interacting with all (3/3) or 2/3 antigens via a shared epitope suggest that the C-terminal stretch containing the epitope might be readily accessible (not structurally buried) and immunogenic at the same time. Other regions on the three Reps might not be immunogenic on a competitive scale. However, also side-groups from other regions of the protein or tertiary structure determinants might affect interaction. The shared C-terminal epitope for mAb22 and mAb23 mirrors previous observations for mAb3 (and even mAb9), sharing an epitope toward the C-terminus (but with a different epitope sequence) and might suggest general accessibility of the C-terminus of Rep proteins from different isolates.

The human (hAb3) and rabbit (rAb3) recombinant version of mAb3 displayed the same reactivity profile as the cognate mouse antibody, representing a big advance with regard to in vivo and clinical applications. In addition, the observed around one log higher sensitivity might support the superior performance of recombinant antibodies, with well-defined heavy and light chain composition and no dynamic drift of antibody-producing hybridoma clonal subpopulations or clonal mixing but might also be affected by different secondary antibody readout systems (rabbit/human vs. mouse).

Comparison between antibody binding data generated with antigens formulated in NaCa and such with antigens in PBS showed an almost perfect correlation (*p* < 0.0001, *r* = 0.99), confirming robustness and reproducibility of the detection, and indicated comparable binding intensity under the two buffer conditions, independent of a pH range from neutral and alkalic. Taken together, nine of the 20 antibodies tested (mAb1, 3, 4, 6, 7, 9, 10, hAb3 and rAb3) bound to the WH2 + C-terminal Rep domain of H1MSB.1, confirming previous epitope mappings (Bund et al. 2021) and suggesting that the C-terminus of (H1MSB.1) Rep represents an immunodominant region targeted by multiple monoclonal antibodies.

To demonstrate the specificity of anti-BMMF Rep antibody reaction against BMMF Rep antigens, we tested cross-reactivity of the antibodies against lysates from a broad range of bacteria present during milk processing. The repertoire of bacterial lysates tested for cross-interaction with anti-BMMF Rep antibodies originated from bacteria that were actually retrieved during milk processing and included milk-associated bacteria (*P. aeruginosa* (Desmousseaux et al. 2025), *B. vesicularis* (Hantsis-Zacharov and Halpern 2007), *P. fluorescens* (Ahmed AH 2024)), mastitis-associated bacteria (*A. viridans* (Saishu et al. 2015), *A. junii/Iwolffii* (Chen et al. 2024; Nam et al. 2009), *A. hydrophila*, *E. faecium* (Kim et al. 2022), *K. oxytoca* (Massé et al. 2020), *S. chromogenes* (De Visscher et al. 2017), *P. putida* (Mallick et al. 2025)) and generally more ubiquitous bacteria (*A. baumannii* (Mohamed et al. 2022), *Bacillus* spp*.*, *S. epidermidis* (Petzer et al. 2022), and *S. paucimobilis* (Vacheyrou et al. 2011)). For a more comprehensive review on bacteria and microbiota in milk, refer to Quigley et al. (2013) or Parente et al. (2020).

Among these bacteria, Rep sequences found on plasmids associated with *A. baumannii* and *lwoffii* (RefSeq) exhibit substantial similarity to Reps of BMMF1 isolates: 88% Rep amino acid sequence homology to H1MSB.1 Rep (*A. baumannii*) and 94% to C1MI.3M.1 Rep (*A. lwoffii*)*,* (Table 2, Supplemental Fig. S2). This prompted us to assess whether BMMF antibodies might cross-detect Reps potentially present in these and other strains. However, none of the six antibodies tested—mAb1, 3, 4, 11, 20, and 22—showed detectable cross-reactivity with the corresponding bacterial lysates. This lack of cross-detection may reflect i) the lack of interaction of the antibodies with the antigens under the given experimental setup, ii) a minimal overlap between the antibody-recognition epitopes of BMMF Reps and those of classical bacterial plasmid Reps, or, iii) the absence of relevant Rep-encoding plasmids (and therefore Rep protein) in the specific bacterial lysates used. The presence or absence of such auxiliary plasmids, including those encoding BMMF-like Rep proteins, is considered highly dynamic in *Acinetobacter*, *Pseudomonas* and other bacteria, as their plasmidomes are shaped by a combination of vertical inheritance and extensive horizontal gene transfer (Maslova et al. 2022; Salgado-Camargo et al. 2020; Virolle et al. 2020). Consequently, strains classified as plasmid-positive in genomic datasets may not stably maintain these plasmids under culture conditions at different test sites. Assuming our test is functional, the observation that even the mAb1 antibody displaying the broadest reactivity (detecting all five BMMF antigens used in this study) did not react with any bacterial lysate argues against a high prevalence of BMMF Rep in the bacterial material; however, transfer of the results of these six tested antibodies to other antibodies is not possible and has to be assessed individually.

We also tested reactivity of anti-BMMF Rep antibodies against commercial purified recombinant *E. coli* RepA (14% amino acid sequence similarity to H1MSB.1 Rep) or against *E. coli* SoluBL21 lysate as outgroups and identified no interaction with the anti-BMMF Rep antibodies. This demonstrates that the antibodies recognize sequence-specific determinants within BMMF Rep proteins rather than e. g. structural features which might be common to plasmid Reps on a broader level. In addition, no cross-reactivity was detected against mammalian samples, including bovine sera, casein, albumin, as well as sera from human, donkey, rabbit, mouse, horse, and pig and human cell lysate. To exclude sample- and matrix-related effects, lysates and sera were spiked with BMMF Rep antigen for the corresponding antibody. No inhibitory effects on detection of the spiked Rep were observed in bacterial lysates and with *E. coli* host proteins, except for mAb20 likely due to limitations in quality. These results underline the high specificity of the BMMF Rep antibodies, which is essential for both monoclonal antibody development (EMA 2016) and downstream applications such as biomarker analyses in patient samples.

Finally, competitive binding assays confirmed the specificity of a representative set of anti-BMMF Rep antibodies during antigen recognition and showed a reduction of the signal by at least 50% when using a 50-fold molar excess of antigen (similarly to Behroozi et al. (2025)), except in the case of mAb22, 1 and 15, which required a 250-fold molar excess. Additionally, no inhibition with *E. coli* SoluBL21 lysate was demonstrated (except for mAb20: 90% inhibition). This is in line with the impurity, mainly originating from the host cell protein (*E. coli* SoluBL21) and aggregation status of the mAb20.

Although most antibodies tested showed similar performance when compared with commercial controls and references, certain antibodies—such as mAb20—require a more considerate handling. MAb20 exhibited a larger hydrodynamic size, a lower purity, and reduced antibody binding sensitivity as well as an unspecific inhibition with *E. coli* SoluBL21 lysate. Although the antibody meets the specificity criterion and detects only C1HB.4 Rep, batch-to-batch variation should be carefully monitored before use. Indeed, mAb21 may serve as a more reliable alternative. Overall, assessments of purity, antibody binding sensitivity and monodispersity is essential for newly generated preparations before application.

In summary, our findings demonstrate that anti-BMMF Rep antibodies can specifically and robustly discriminate between the Reps of closely related BMMF1 isolates and did not show off-target binding to sequence- or structurally-related bacterial or mammalian proteins. This specificity generally supports the use of the antibodies for exploring the potential role of BMMFs as biomarkers in cancer biology e. g. in diagnostic immunohistological or serological approaches. Future antibody application will still require individual validation of specificity for each antibody including inhibition testing of matrix. Therapeutic application likely requires increased antibody quality, which can be achieved via standardized antibody production and further purification protocols, as suggested above. The anti-BMMF Rep antibody binding assay developed in this study may also be used in an ELISA platform for detecting BMMF antigens in clinical specimens. In addition, recombinant rabbit and humanized versions of the lead antibody mAb3 are now available, which upon individual validation in the respective readouts allow diagnostic or therapeutic application in mouse models as well as in human in vitro and in vivo studies.

## Supplementary Information

Below is the link to the electronic supplementary material.

ESM 1 (DOCX 549 KB)

## Data Availability

The data of the current study is available from the corresponding author upon reasonable request.
