# Supplementary material for: Characterization of antibodies against the replication protein (Rep) encoded by bovine meat and milk factors (BMMFs)
Source: Appl Microbiol Biotechnol. 2026 Apr 16;110:135. doi: 10.1007/s00253-026-13809-x (PMC13099706; doi:10.1007/s00253-026-13809-x)
Supplement: Supplementary file 1 [file 253_2026_13809_MOESM1_ESM.pdf]

Supplementary Material

Journal:

Applied Microbiology and Biotechnology

Title:

Characterization of Antibodies Against the Replication Protein (Rep) Encoded by Bovine Meat and Milk Factors (BMMFs)

Veronika Frehtman*[1], Gunjan Shukla*[1], Michael Gentz[1], Marcus Müller[1], Oladimeji Paul Duduyemi[1], Imke Grewe[1], Claudia Ernst[1], Claudia Tessmer[1], Andrea Didier[2], Ilse Hofmann[1], Timo Bund*‡[1], Barbara Leuchs*‡[1]

[1]German Cancer Research Center, Im Neuenheimer Feld 280, 69120 Heidelberg, Germany

[2]Faculty of Veterinary Medicine, Ludwigs Maximilians University Munich, Schönleutnerstraße 8, 85764 Oberschleißheim, Germany

* Authors contributed equally

‡ Shared corresponding authorship

Corresponding authors:

Barbara Leuchs
German Cancer Research Center
Biopharmaceutical Processing and Development Unit
Im Neuenheimer Feld 280
69120 Heidelberg
Germany
B.Leuchs@dkfz.de; +49 6221 424300

Dr. Timo Bund
German Cancer Research Center
Episomal-Persistent DNA in Cancer- and Chronic Diseases
Im Neuenheimer Feld 280
69120 Heidelberg
Germany
t.bund@dkfz-heidelberg.de; +49 6221 424655

Supplemental Table S1: Binding reactivity (absorption A_{450nm}-A_{595nm} measured with anti-BMMF Rep antibody binding assay) of anti-BMMF Rep antibodies to BMMF Rep proteins and peptides formulated either in sodium-carbonate buffer (NaCa), phosphate buffer (PBS) or water for injection (WFI).

**Antigen**	**H1MSB.1 Rep**		**H1MSB.1 WH1 Rep**		**H1MSB.1 WH1+WH2 Rep**		**H1MSB.1 WH2+C-term Rep**		**H1MSB.2 Rep**		**C1HB.4 Rep**		**C1MI.3M.1 Rep**		**C1MI.9M.1 Rep**		**Rep peptide 1**	**Rep peptide 2**	**Rep peptide 3**	***E. coli* RepA**
**Formulation**	**NaCa**	**PBS**	**NaCa**	**PBS**	**NaCa**	**PBS**	**NaCa**	**PBS**	**NaCa**	**PBS**	**NaCa**	**PBS**	**NaCa**	**PBS**	**NaCa**	**PBS**	**WFI**	**WFI**	**WFI**	**PBS**
**mAb1**	1.9	2.0	0.0	0.0	1.9	2.1	1.8	1.9	2.0	2.1	0.8	1.2	1.3	1.5	2.3	1.8	0.0	2.0	0.0	0.0
**mAb2**	0.7	0.6	0.6	0.8	0.4	1.0	0.0	0.0	0.9	1.1	0.0	0.0	0.0	0.0	0.9	0.3	0.6	0.0	0.0	0.0
**mAb3**	0.9	1.0	0.0	0.0	0.0	0.0	0.8	1.0	0.0	0.0	0.0	0.0	0.0	0.0	0.0	0.0	0.0	0.0	0.0	0.0
**hAb3**	1.1	1.4	0.0	0.0	0.0	0.1	1.1	1.3	0.0	0.0	0.0	0.0	0.0	0.0	0.0	0.0	-0.1	-0.1	0.0	0.0
**rAb3**	1.6	1.6	0.1	0.0	0.0	0.0	1.4	1.5	0.0	0.1	0.2	0.1	0.2	0.1	0.1	0.0	-0.1	-0.1	-0.1	0.1
**mAb4**	0.9	1.2	0.0	0.0	0.0	0.0	0.7	1.1	0.0	0.0	0.1	0.0	0.0	0.0	0.0	0.0	0.0	0.0	0.0	0.0
**mAb5**	0.7	1.2	1.4	1.5	0.6	1.5	0.0	0.0	1.2	1.5	0.0	0.0	0.2	0.1	1.5	0.9	1.0	0.0	0.0	0.0
**mAb6**	0.6	0.9	0.0	0.0	0.1	0.1	0.4	0.7	0.0	0.0	0.0	0.0	0.0	0.0	0.0	0.0	0.0	0.0	0.0	0.0
**mAb7**	0.6	0.7	0.3	0.3	0.1	0.2	0.5	0.6	0.1	0.2	0.1	0.1	0.1	0.1	0.0	0.0	0.0	0.0	0.0	0.0
**mAb8**	0.8	0.8	1.1	1.1	0.9	1.0	0.0	0.0	0.0	0.0	0.0	0.0	0.0	0.0	0.0	0.0	0.0	0.0	0.0	0.0
**mAb9**	1.0	1.2	0.1	0.2	0.0	0.2	1.0	1.2	0.1	0.2	0.0	0.0	0.0	0.0	0.0	0.0	0.0	0.0	0.0	0.0
**mAb10**	1.1	1.2	0.1	0.1	0.1	0.1	1.0	1.1	0.0	0.0	0.0	0.0	0.0	0.0	0.0	0.0	0.0	0.0	0.0	0.0
**mAb11**	1.4	1.4	1.6	1.5	1.6	1.6	0.0	0.0	1.4	1.4	0.0	0.0	0.9	0.9	1.3	1.0	0.0	0.0	0.0	0.0
**mAb13**	1.1	1.0	1.3	1.3	1.2	1.3	0.0	0.0	0.6	0.6	0.0	0.0	0.1	0.1	0.3	0.1	0.0	0.0	0.0	0.0
**mAb14**	0.5	1.1	1.3	1.4	0.6	1.4	0.0	0.0	1.1	1.4	0.0	0.0	0.1	0.1	1.4	0.8	0.9	0.0	0.0	0.0
**mAb15**	0.6	1.0	1.3	1.4	0.7	1.4	0.0	0.0	1.1	1.3	0.0	0.0	0.1	0.1	1.6	1.0	0.9	0.0	0.0	0.0
**mAb20**	0.0	0.0	0.0	0.0	0.0	0.0	0.0	0.0	0.0	0.0	0.7	0.6	0.2	0.0	0.0	0.0	0.0	0.0	0.0	0.2
**mAb21**	0.0	0.0	0.0	0.0	0.0	0.0	0.0	0.0	0.0	0.0	0.6	0.7	0.0	0.0	0.0	0.0	0.0	0.0	0.0	0.0
**mAb22**	0.0	0.0	0.0	0.0	0.0	0.0	0.0	0.0	1.9	2.0	0.0	0.0	1.8	1.7	2.0	1.5	0.0	0.0	1.1	0.0
**mAb23**	0.0	0.0	0.0	0.0	0.0	0.0	0.0	0.0	1.0	1.2	0.0	0.0	0.3	0.4	0.2	0.0	0.0	0.0	0.0	0.0
**anti-penta His**	0.9	0.9	0.9	0.8	0.7	0.7	0.8	0.9	0.7	0.9	0.4	0.4	0.6	0.4	0.6	0.5	0.0	0.0	0.0	0.0
**mouse IgG1 isotype control**	0.0	0.0	0.0	0.0	0.0	0.0	0.0	0.0	0.0	0.0	0.0	0.0	0.0	0.0	0.0	0.0	0.0	0.0	0.0	0.0
**anti-*E. coli* RepA**	n.a.	n.a.	n.a.	n.a.	n.a.	n.a.	n.a.	n.a.	n.a.	n.a.	n.a.	n.a.	n.a.	n.a.	n.a.	n.a.	n.a.	n.a.	n.a.	1.1

Antibody reactivity was defined for binding intensities exceeding 0.3 (A_{450nm}-A_{595nm})

Supplemental Table S2: Test for cross reactivity of bacterial lysates with selected anti-BMMF Rep antibodies.

		Antibodies	mAb1	mAb3	mAb4	mAb11	mAb20	mAb22
	**Analyte**	**Milk tank / MHI no.**	**H1MSB.1 Rep**				**C1HB.4 Rep**	**C1MI.3M.1 Rep**
**PC**	**H1MSB.1 Rep**		+++	+++	+++	+++		
	**C1HB.4 Rep**						+++	
	**C1MI.3M.1 Rep**							+++
**NC**	**PBS**		-	-	-	-	-	-
**Bacterial lysate**	***Acinetobacter baumannii***	**#42**	-	-	-	-	-	-
	Acinetobacter junii/lwoffii	**#52**	-	-	-	-	-	-
	Acinetobacter lwoffii	**#44**	-	-	-	-	-	-
	Aerococcus viridans	**#45**	-	-	-	-	-	-
		**#47**	-	-	-	-	-	-
		**#57**	-	-	-	-	-	-
		**#58**	-	-	-	-	-	-
	Aeromonas hydrophila	**MHI 1004**	-	-	-	-	-	-
	***Bacillus* spp**	**#46**	-	-	-	-	-	-
	Brevundimonas vesicularis	**#48**	-	-	-	-	-	-
		**#77**	-	-	-	-	-	-
	Enterococcus faecium	**#50**	-	-	-	-	-	-
	Klebsiella oxytoca	**#51**	-	-	-	-	-	-
	***Pantoea* spp**	**#116**	-	-	-	-	-	-
	Pseudomonas aeruginosa	**MHI 1000**	-	-	-	-	-	-
		**MHI 1018**	-	-	-	-	-	-
	Pseudomonas fluorescens	**MHI 1002**	-	-	-	-	-	-
	Pseudomonas putida	**MHI 1017**	-	-	-	-	-	-
	Sphingomonas paucimobilis	**#103**	-	-	-	-	-	-
	Staphylococcus chromogenes	**#53**	-	-	-	-	-	-
	Staphylococcus epidermidis	**#43**	-	-	-	-	-	-

Bacteria for lysate preparations were either isolated from the bulk tank of the LMU research and teaching farm (milk tank number #) or derived from the Chair's in house strain collection (Milkhygiene Institute: [MHI] internal sample reference number). Bacteria were cultured until the stationary phase was reached. Antibody reactivity with bacterial lysates is defined as the antibody binding signal as follows: <0.3: - ; ≥0.3<0.5: + ; ≥0.5<1: ++ ; ≥1: +++ . Positive controls (PC) and negative controls (NC) were included in parallel to prove consistency of detection

Supplemental Table S3: Assessment of possible inhibitory effects of the mammalian specimens and bacteria lysates characterized by spiking with a defined amount (200 ng/well) of BMMF Rep antigen prior to coating and comparison to non-spiked controls measured with anti-BMMF Rep antibody binding assay.

			Antibody			
	**Specimen details**		**mAb 1**	**mAb 3**	**mAb 20**	**mAb 22**
**Antibody binding signals**	**PC**	**H1MSB.1 Rep**	2.0	1.3		
		**C1HB.4 Rep**			1.1	
		**C1MI.3M.1 Rep**				2.2
	**NC**	**Buffer**	0.0	0.0	0.0	0.0
			**Spiked with Ag**			
			**H1MSB.1 Rep**		**C1HB.4 Rep**	**H1MSB.2 Rep C1MI.3M.1 Rep**
**Spike recoveries %**	**Bovine**	**Fetal sera**	75	41	8	78
		**Casein**	100	77	59	96
		**Serum albumin**	99	78	50	96
	**Human**	**HEK cell lysate**	72	72	33	95
		**Human sera**	53	37	10	94
	**Diverse Mammalian**	**Donkey sera**	50	30	15	78
		**Rabbit sera**	72	47	14	86
		**Mouse sera**	15	20	0	43
		**Horse sera**	64	38	19	85
		**Pig sera**	53	35	13	83
	***E. coli* SoluBL21**	**Cell lysate**	91	87	36	103
	**Bacteria lysis buffer spiked with PC**	**with protease inhibitor**	88	95	100	100
		**with Lysozyme**	94	97	100	96
	Acinetobacter baumannii	**#42**	90	82	98	90
	Acinetobacter junii/lwoffii	**#52**	86	93	103	86
	Acinetobacter lwoffii	**#44**	87	94	98	91
	Aerococcus viridans	**#45**	64	86	99	76
		**#47**	87	91	106	94
		**#57**	62	85	93	83
		**#58**	87	91	102	95
	Aeromonas hydrophila	**MHI 1004**	91	100	101	95
	***Bacillus* spp**	**#46**	56	82	82	82
	Brevundimonas vesicularis	**#48**	85	99	92	88
		**#77**	92	93	107	94
	Enterococcus faecium	**#50**	88	98	109	94
	Klebsiella oxytoca	**#51**	90	93	102	96
	***Pantoea* spp**	**#116**	89	91	105	92
	Pseudomonas aeruginosa	**MHI 1000**	82	79	96	91
		**MHI 1018**	89	90	99	90
	Pseudomonas fluorescens	**MHI 1002**	97	81	85	91
	Pseudomonas putida	**MHI 1017**	51	61	57	61
	Sphingomonas paucimobilis	**#103**	84	94	96	92
	Staphylococcus chromogenes	**#53**	86	98	103	98
	Staphylococcus epidermidis	**#43**	87	92	90	93

Antibody binding signals (A_{450nm}-A_{595nm}) for positive controls (PC) and system negative controls (NC) are given as a reference. The spike recoveries (%) of the respective antibody antigen combinations were measured in mammalian specimens and bacterial lysates. For the primary antibodies mAb1 and mAb3, the samples were spiked with H1MSB.1 Rep, for mAb20 with C1HB.4 Rep. For mAb22, bovine, human, diverse mammalian and E. Coli samples were spiked with the H1MSB.2 Rep antigen while milk bacteria lysate samples were spiked with C1MI.3M.1 Rep

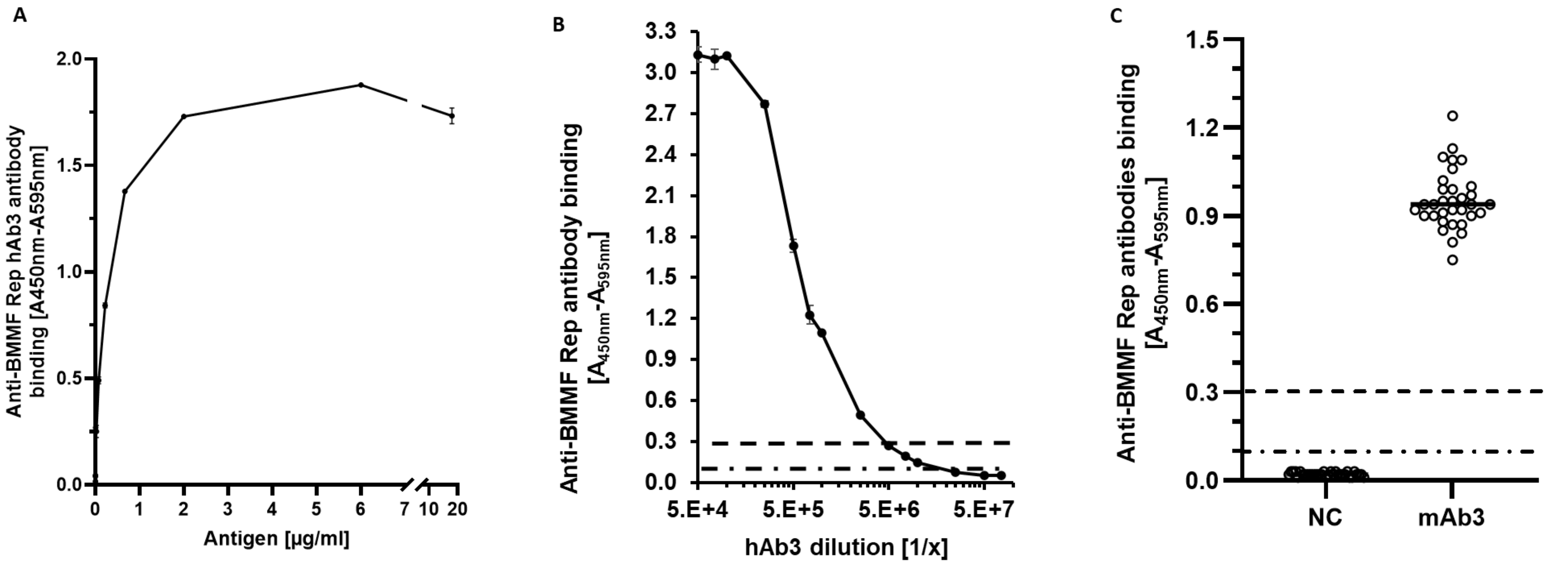

Supplemental Fig. S1: Anti-BMMF Rep antibody binding assay development. (A) HAb3 binding to different concentrations of H1MSB.1 Rep antigen. (B) Representation of the range of the linear binding signal using a serial dilution of hAb3 for binding to H1MSB.1 Rep. (C) Definition of background (NC) cutoff <0.1 A_{450nm}-A_{595nm} (dashed-dotted lines) and reactivity cutoff ≥ 0.3 A_{450nm}-A_{595nm} (dashed lines) based on 33 individual measurements of mAb3 binding to H1MSB.1 Rep (used as technical control) or buffer (NC) for the calculation of coefficient of variation (CV = 10%)

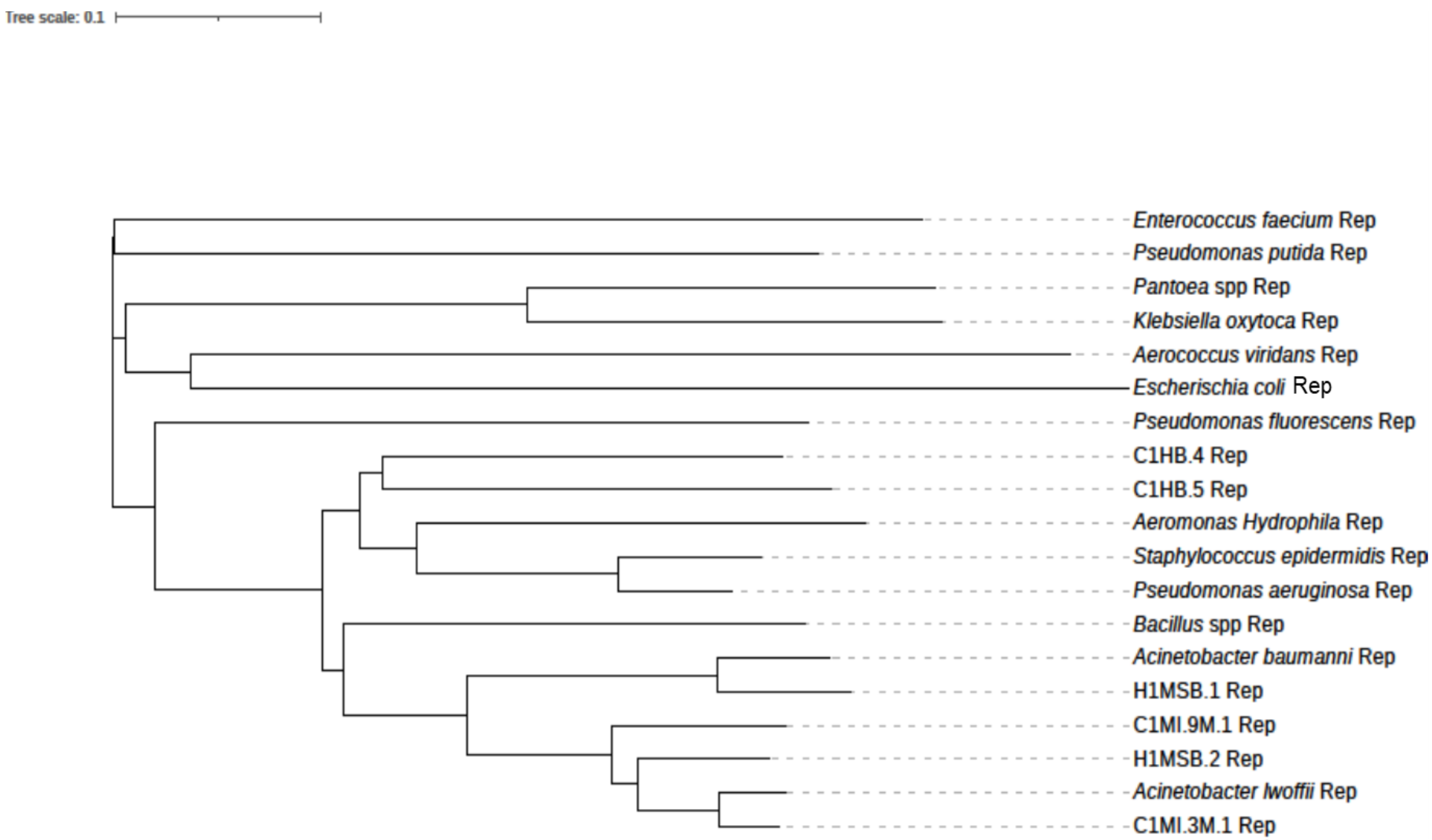

Supplemental Fig. S2: Phylogenetic tree generated with Clustal Omega illustrating the evolutionary relationships among Rep proteins of BMMF isolates and reference Rep proteins from bacterial species associated with milk processing environments. *E. coli* Rep A was included as an outgroup. Reference Rep representatives were selected based on the closest homologs to H1MSB.1 Rep identified in the NCBI Protein Reference Sequence (RefSeq) database to capture the diversity of Rep proteins within the bacterial plasmidome (tree modified with iTOL V 7.5)
